# Influence of adaptive capacity on the outcome of climate change vulnerability assessment

**DOI:** 10.1038/s41598-017-13245-y

**Published:** 2017-10-11

**Authors:** Benjamin Y. Ofori, Adam J. Stow, John B. Baumgartner, Linda J. Beaumont

**Affiliations:** 1 Department of Biological Sciences, Macquarie University, North Ryde, Macquarie Park, NSW 2019 Australia; 2 Department Animal Biology and Conservation Science, University of Ghana, Legon-Accra, Ghana

## Abstract

Climate change vulnerability assessment (CCVA) has become a mainstay conservation decision support tool. CCVAs are recommended to incorporate three elements of vulnerability – exposure, sensitivity and adaptive capacity – yet, lack of data frequently leads to the latter being excluded. Further, weighted or unweighted scoring schemes, based on expert opinion, may be applied. Comparisons of these approaches are rare. In a CCVA for 17 Australian lizard species, we show that membership within three vulnerability categories (low, medium and high) generally remained similar regardless of the framework or scoring scheme. There was one exception however, where, under the warm/dry scenario for 2070, including adaptive capacity lead to five fewer species being classified as highly vulnerable. Two species, *Eulamprus leuraensis* and *E*. *kosciuskoi*, were consistently ranked the most vulnerable, primarily due to projected losses in climatically suitable habitat, narrow thermal tolerance and specialist habitat requirements. Our findings provide relevant information for prioritizing target species for conservation and choosing appropriate conservation actions. We conclude that for the species included in this study, the framework and scoring scheme used had little impact on the identification of the most vulnerable species. We caution, however, that this outcome may not apply to other taxa or regions.

## Introduction

Climate change poses a substantial threat to global biodiversity. Prioritizing conservation actions to ameliorate the impacts of climate change requires that we assess the vulnerability of species to climate change and identify which traits or characteristics drive their vulnerability^1–3^. To this end, climate change vulnerability assessment (CCVA) has become a mainstay conservation decision support tool^4–8^. However, most do not assess all the elements of vulnerability and hence paint an incomplete picture of a species’ vulnerability to climate change^8,9^.

The vulnerability of a population, species or community is considered a function of three elements: exposure, sensitivity and adaptive capacity^3,4,10^. Exposure is the magnitude of climate change likely to be experienced by a species across its range, and depends on the rate and magnitude of climate change^10^. It is typically quantified using species distribution models (SDMs) fitted with climate variables, and projected onto downscaled output from regional or global circulation models (RCMs, GCMs)^10,11^. Most studies have measured exposure using projected change in a species’ climatically suitable habitat, overlap between the current and future suitable habitat and projected loss of suitable habitat within protected areas^12–15^. Other studies have also included changes in food availability, extreme weather and sea level^16,17^.

Sensitivity is the degree to which the performance, survival and persistence of species is affected by climate change^10^. Most CCVAs have assessed sensitivity using life-history traits, including physiological, behavioural or ecological traits, such as tolerance to temperature or hydrological regimes, habitat specificity and dietary specialization, occupied area, population size, reproductive rate, temperature-dependent sex determination, clutch size, growth rate, generation length, and life span^3,7,10,11,13,15,18^. In general, the number and combination of life-history traits employed in CCVAs varies with the study species, their habitats and data availability^5^.

Adaptive capacity is the potential for species or populations to tolerate or adapt to climate change^10^. Like sensitivity, adaptive capacity is governed by intrinsic traits, but can be influenced by extrinsic traits, such as habitat loss and fragmentation^19^. Although the distinction between sensitivity and adaptive capacity is somewhat ambiguous, dispersal and colonization ability, microevolution and phenotypic plasticity are generally regarded as the components of adaptive capacity^10,19^. Dispersal allows organisms to move to regions with suitable habitat^20,21^. It also promotes gene flow that increases genetic diversity, fitness and evolutionary potential of geographically isolated populations^22^. However, it is unclear whether the natural dispersal rates of species, particularly philopatric species, will be sufficient to track the movement of climate zones^23,24^. Also, anthropogenic and natural barrier to dispersal (e.g., roads, large water bodies, mountain ranges), threatening processes (e.g., predation, disease, hunting) in the landscape and aspects of climate that affect dispersal capacity may prevent organisms from tracking their climate niche^25–27^.

Evolutionary adaptive capacity is the ability for species or populations to adapt *in situ* through micro-evolution^28^. Evolution is a change in allele frequency, and thus requires adequate heritable genetic variation in populations^29,30^. Although the rate of evolutionary adaptation varies among species and populations, and in space and time^28^, it occurs faster in species with high genetic diversity, large population size, high fecundity and short generation time^31^. For many species, it remains unclear if they can adapt at a sufficient speed to counter the projected rate of climate change. Recent studies, however, have suggested that microevolution may be more rapid than previously thought^28,32^.

In addition to evolutionary adaptation, species may exhibit phenotypic plasticity, whereby individuals change their phenology, physiology or morphology without undergoing changes in their genetic makeup^33^. Individuals have greater fitness when their phenotypes suit the environment^34,35^, yet as climate changes, the phenotype and phenology of populations may no longer confer high fitness^35,36^. Although phenotypic plasticity alone may not be sufficient for the long-term persistence of species under rapid climate change^37^ and in fact, may buffer selection and slow evolution (i.e., the Baldwin effect)^29,38^, it can increase the rate of evolution or buy time for evolutionary adaption^35^.

The components of adaptive capacity can be assessed by empirical, observational and modelling studies^10^. Yet, for most species, there is very little available information on dispersal rates, evolutionary capacity and phenotypic plasticity, or the thresholds at which they are considered adequate to counter the impacts of climate change^19^. Therefore, like sensitivity, adaptive capacity is relative and a better understanding of the contributions of its components to a species’ resilience and resistance to climate change is crucial for advancing its quantification^3^.

A robust CCVA should account for all three elements, thereby facilitating identification of the most vulnerable species and the characteristics that determine their vulnerability^8,10^. However, recent reviews indicate that CCVAs are commonly based on sensitivity and exposure^9,39^. It has been suggested that assessments that fail to account for any of the three elements may be incomplete and produce biased outcomes, thereby rendering them less reliable for guiding conservation decisions^4,8,15^.

Another difference in the approach to CCVA is whether all traits incorporated into the analysis contribute equally to a species’ vulnerability score. Both unweighted and weighted scoring schemes (where the latter attempts to capture the perceived relative contribution of the various traits to vulnerability) have been used e.g.^13–15,40^, but the outcome of these scoring systems have been rarely compared.

Here, we adopted and extended existing CCVA frameworks to include the capacity of species to adapt to climate change and explored the different approaches to undertaking CCVAs. Our main objective was to assess the relative vulnerability of 17 lizard species distributed partly or wholly along the Great Dividing Range of southeastern Australia, and the factors that make them susceptible to climate change. In doing so, we evaluated the extent to which the omission of adaptive capacity in CCVA, and the weighting of scoring schemes, influences the assessment outcome. Specifically, we asked the following questions: (i) Which species are most vulnerable to climate change and what factors are responsible for their vulnerability? (ii) Does the omission of adaptive capacity influence the outcome of CCVAs? (iii) What effect does unweighted and weighted systems have on the outcome of CCVAs? We hypothesize that the number and composition of species ranked as highly vulnerable could change when adaptive capacity is included in the CCVA. We also expect the weighted system to influence the composition and order of species listed under different vulnerability categories.

## Results

### Exposure

**Figure 1:**
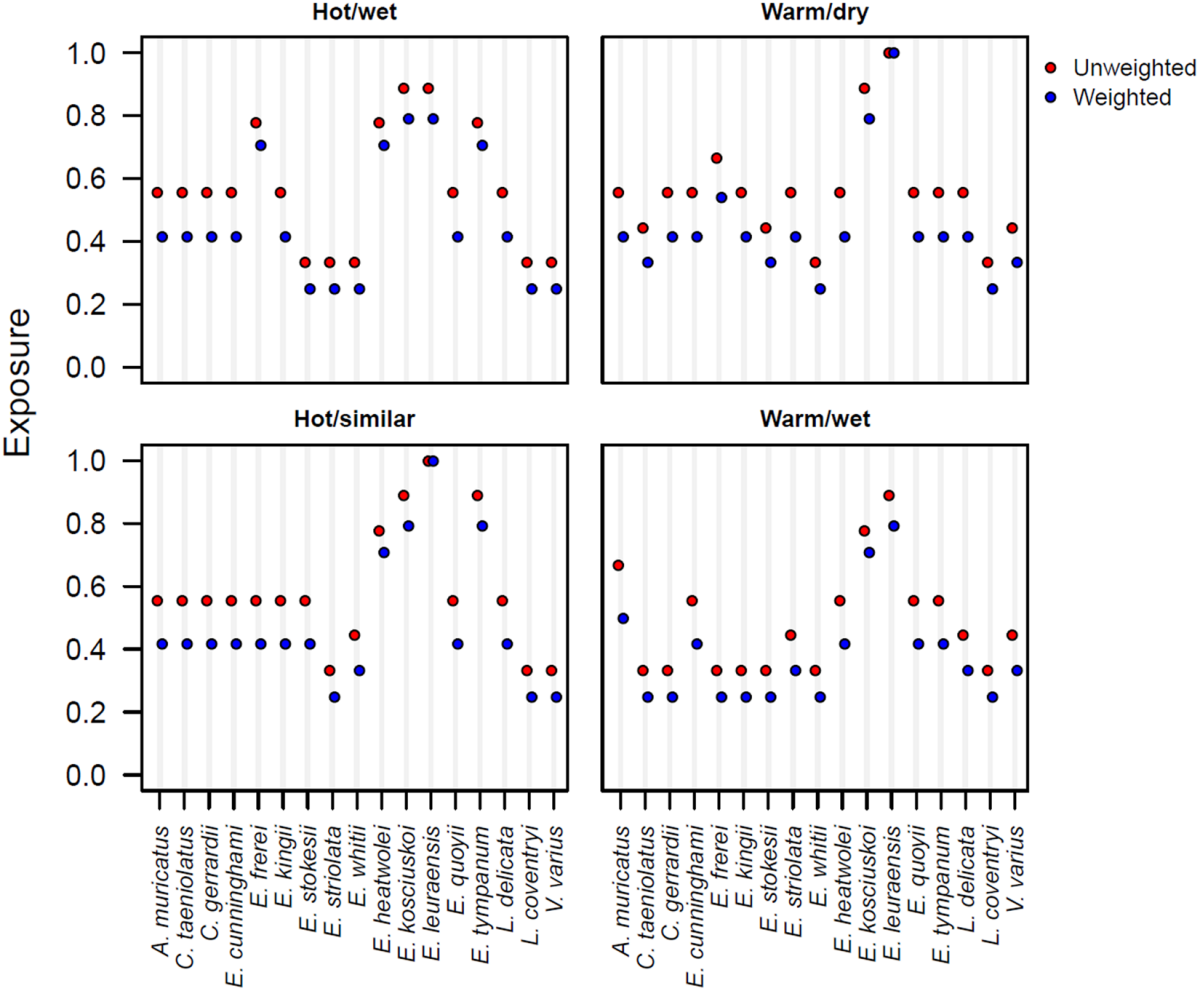
Weighted and unweighted exposure scores for 17 Australian lizards under projected climate change by 2050. Exposure was assessed using four contrasting future climate scenarios that are equally plausible: hot/wet; warm/dry; hot/similar precipitation; and warm/ wet scenarios. Score ≥0.67 is high, from 0.33 to 0.66 is moderate and <0.33 is low. See Supporting Information Figures S1 and S2 for exposure scores at 2030 and 2070.

Loss of suitable habitat varied across climate trajectories and time horizons, with up to 15 lizards (88%) projected to lose portions of their climatically suitable habitat by 2070 (Supporting Information Table S1). Seven species (41%), including *Eulamprus leuraensis*, *Eulamprus kosciuskoi*, *Eulamprus heatwolei*, *Eulamprus tympanum*, *Egernia frerei*, *Egernia kingii* and *Egernia cunninghami* were projected to lose at least 50% of their current suitable habitat by 2070. *Lissolepis coventryi* was the only species projected to gain (26%-136%) climatically suitable habitat under all climate scenarios and time horizons considered (Supporting Information Table S1). The proportion of species assigned to the three exposure categories (low, moderate and high) also varied with climate trajectory and time horizon. In general, most species were assigned to the moderate and low exposure categories (Fig. 1), with no significant difference between the weighted and unweighted scoring schemes (Fisher’s exact test: df = 32; *p* > 0.05). *Eulamprus leuraensis* was the only species assigned to the high exposure category under all the climate trajectories and time horizons considered.

### Sensitivity

**Figure 2:**
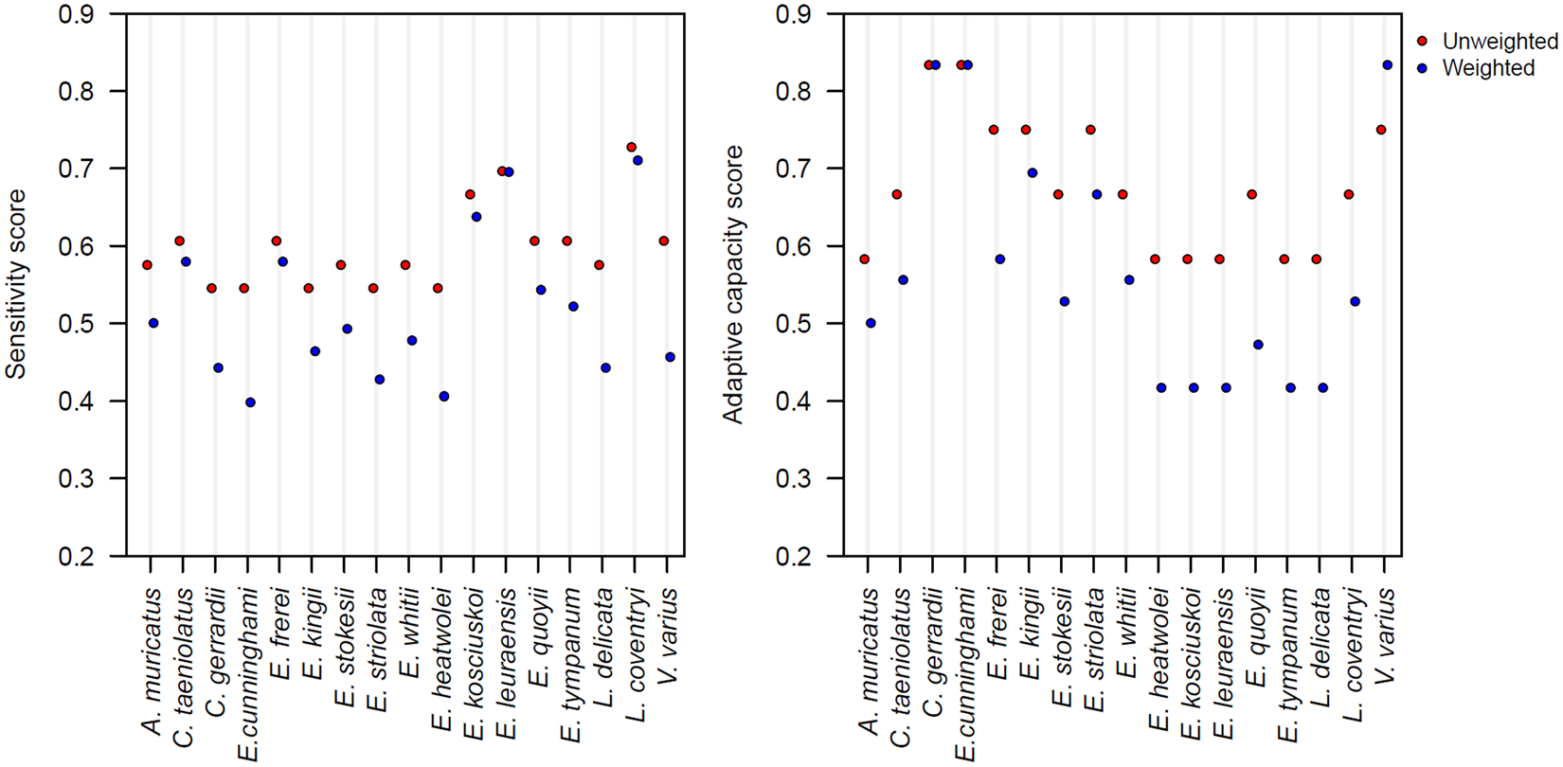
Weighted and unweighted scores of sensitivity and adaptive capacity of the 17 Australian lizard species to climate change. Score ≥0.67 is high, from 0.33 to 0.66 is moderate and <0.33 is low.

Two species were categorised as having high sensitivity to climate change: *E*. *leuraensis* and *L*. *coventryi*, both of which are habitat specialists, have small population sizes and narrow geographic range sizes. The remaining species were assigned to the moderate sensitivity category, with scores between 55–66 for the unweighted score and 40–64 for the weighted score. Sensitivity scores for the two scoring schemes were strongly correlated (Spearman’s correlation, *r*_*s*_ = 0.91, *df* = 32, *p < *0.01), and although the unweighted scores were higher than the weighted scores (Fig. 2) this had no effect on the proportion and identity of the species assigned to the various sensitivity categories.

### Adaptive capacity

None of the 17 species were classified as having low adaptive capacity (Ac), and those with high Ac were characterised by high genetic diversity and dispersal capacity. Depending upon the scoring scheme and time period, up to 76% of the species were assigned to the moderate Ac category. Six species, *Cyclodomorphus gerrardii*, *Varanus varius*, *Egernia striolata*, *E*. *cunninghami*, *E*. *kingii* and *E*. *frerei* were classified as having high Ac under the unweighted scoring scheme. Four of these (excluding *E*. *frerei* and *E*. *striolata*) were also assigned to the high Ac category by the weighted scoring scheme.

### Overall vulnerability

#### Accounting for exposure, sensitivity and adaptive capacity

**Figure 3:**
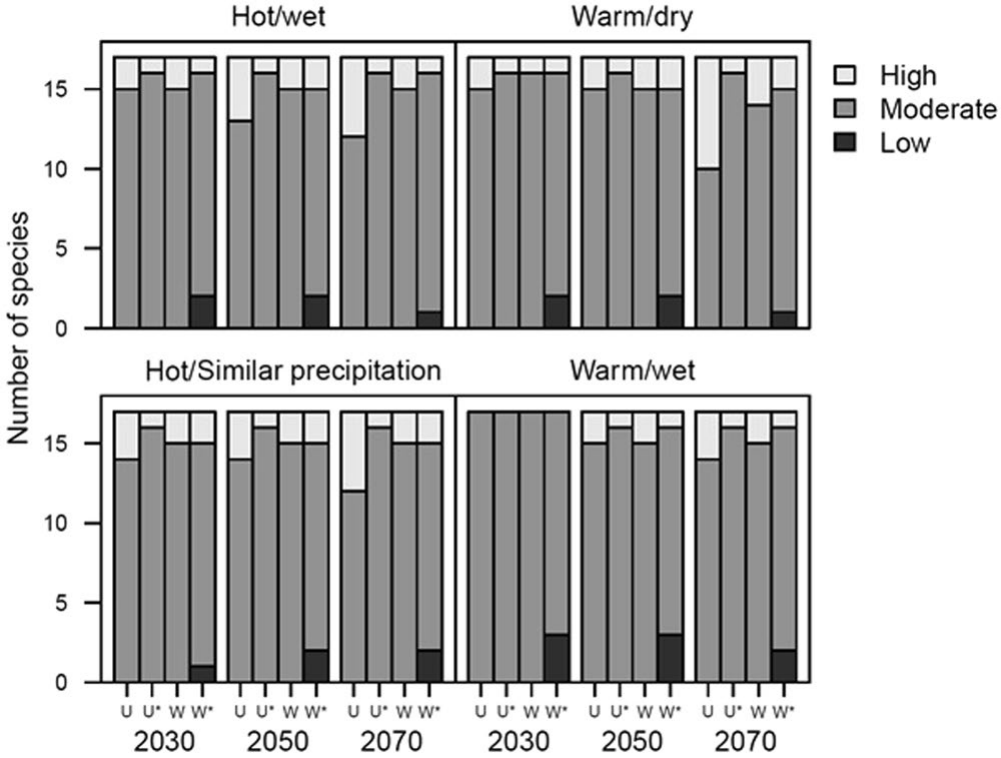
Number of species assigned to the three climate change vulnerability rankings for the 17 lizards, according to the unweighted (U) and weighted (W) scoring schemes. Asterisks (*) indicate analyses that considered all three elements of vulnerability: exposure, sensitivity and adaptive capacity.

Although the vulnerability scores for individual species varied across the four climate scenarios and three time horizons (Supporting Information Table S6), membership within the three vulnerability categories remained relatively similar. Generally, there were no significant differences between the two scoring schemes or between the CCVA with (ES) and without (ESA) adaptive capacity (Fisher’s exact test: df = 32, *p* > 0.05) (Fig. 3). There was an exception, however. In the unweighted scoring scheme, under the warm/dry scenario for 2070, omitting adaptive capacity led to five additional species being classified as highly vulnerable, compared to the two (*E*. *leuraensis* and *E*. *kosciuskoi*) included in the ESA framework. Indeed, *E*. *leuraensis* and *E*. *kosciuskoi* were classified as highly vulnerable in both frameworks and scoring schemes, across most of the climate scenarios and time horizons.

According to the unweighted scheme, *E*. *frerei*, *E*. *cunninghami* and *E*. *kingii* are borderline potential adapters because they are highly exposed under at least one climate scenario and time slice, and have high adaptive capacity, but moderate sensitivity to climate change. Under both unweighted and weighted schemes, *Lissolepis coventryi* is on the borderline of high latent risk because it has low exposure, high sensitivity and moderate adaptive capacity across all the climate scenarios and at least two time slices.

## Discussion

In this era of rapid climate change, making informed decisions about where and how to allocate resources for conservation is crucial^8,41^. Here, we applied a CCVA framework that integrates species’ exposure, sensitivity and capacity for adapting to climate change, to 17 lizard species distributed across the Great Dividing Range of Australia. As hypothesized, we found that incorporating adaptive capacity into a CCVA influenced the composition of species assigned to the various vulnerability categories, although there were exceptions. Our results also highlighted how the degree of exposure to climate change is temporally dynamic, and ought to be assessed over multiple time horizons to facilitate informed, species-specific management decisions. Our findings provide relevant information to guide conservation strategies for Australian lizards under climate change.

When all three elements of vulnerability were integrated, the difference between the number of species within the various vulnerability categories under the weighted and unweighted schemes were not significant (Fisher’s exact test: df = 32; *p* > 0.05). The composition of species within the three vulnerability categories also did not change. The majority (≥59%) of lizard species were classified as moderately vulnerable under both schemes, with only one to two assigned to the high vulnerability category (weighted and unweighted schemes, respectively). The two species, *E*. *leuraensis* and *E*. *kosciuskoi*, were assigned to the high vulnerability category. The key contributors to their vulnerability were substantial losses in the spatial extent of climatically suitable habitat, narrow thermal tolerance, small geographic range, and low dispersal capacity. However, it has been suggested that species assigned to a high vulnerability category may persist if they are able to adapt to the novel future climate^3,10^. Although both *E*. *leuraensis* and *E*. *kosciuskoi* have moderate adaptive capacity, this measure is relative and does not highlight a species’ true capacity to adapt to changes in climate^19^. As such, active monitoring and management of these species might be necessary to prevent climate-driven extinction. Actions should be aimed at reducing their sensitivity to climate change; external stressors, such as habitat loss and degradation, predation by feral cats, and competition with invasive species should be managed. Where there is evidence that their populations are declining over time, assisted migration may be necessary to safeguard the species from extinction.

The species that are not currently at risk from climate change could also be categorized as being potential adapters, potential persisters, or as having high latent risk, depending on their exposure, sensitivity and adaptive capacity rankings. Each of these classes has particular implications for conservation management^3^. Potential adapters are the species that are highly exposed and sensitive to climate change, but have high capacity to adapt and tolerate the impacts of the change^3,10^. Potential persisters are species that are highly exposed and have low adaptive capacity, but are not sensitive to climate change^3,10^. Species in this class may be able to tolerate changes in climate conditions. Species with high latent risk are those with high sensitivity and low adaptive capacity, but are currently not highly exposed to climate change. These species are not of immediate management concern, but could become highly vulnerable if exposed beyond the modelled time frame or if the rate of change in exposure outpaces that in the GCMs that we used^3,10^.

According to the unweighted scheme, *E*. *frerei*, *E*. *cunninghami* and *E*. *kingii* are highly exposed under at least one climate scenario and time slice, and have high adaptive capacity, but moderate sensitivity to climate change. These species are therefore borderline potential adapters and must be monitored to ensure they retain stable populations over time. Under both schemes, *Lissolepis coventryi* has low exposure, high sensitivity and moderate adaptive capacity across all the climate scenarios and at least two time slices. This species lies at the border of high latent risk and not of immediate conservation concern. Species that are highly exposed, but have moderate sensitivity and adaptive capacity, such as *E*. *leuraensis*, *E*. *tympanum* are also not of immediate concern, but represent new priorities for conservation. Changes to external stressors, such as habitat degradation and high predation, competition and disease, which increase their sensitivity and decrease their adaptive capacity may result in these species becoming more vulnerable to climate change.

The species assigned to the high exposure category are projected to experience greater losses in climatically suitable habitat than those assigned to the moderate and low categories. This supports the findings of other studies on Australian reptiles^13,14^ and suggests that loss in climatically suitable habitat will be a key driver of the vulnerability of lizards in Australia. For species with sufficient dispersal capabilities, creating and maintaining connectivity between current and future habitat may be necessary to facilitate their movement across the landscape^42,43^. Connecting populations that are in close proximity, but are separated by physical barriers, could also enhance gene flow, genetic diversity and the capacity of species to adapt to climate change.

A frequent challenge to CCVAs has been the lack of data on adaptive capacity^8,19^. Yet, we demonstrate that ignoring adaptive capacity can influence the results; more species were classified as highly vulnerable, especially under the weighted scheme. This may impede reliable identification of conservation priorities, leading to suboptimal use of limited conservation resources. This emphasizes the need to comprehensively assess all three elements of vulnerability where such data are available, as advocated by recent studies^3,8^.

Our study identified only two species as highly vulnerable to climate change. However, these results should be interpreted with caution, because we only considered 17 species in our analysis, and it is possible that we excluded several species that are highly vulnerable to climate change. For example, the montane specialist skink *Lampropholis robertsi* and its congener *L*. *coggeri* are endemic to rainforests of northeast Australia, have relatively narrow distributions, occur within a narrow climatic zone, and have relatively low dispersal capacity. These characteristics suggest they might be highly vulnerable to climate change, but they were not included in our analysis because their occurrence records were too few to allow for reliable models.

Like exposure and sensitivity, adaptive capacity scores are relative and are sensitive to the kind and number of variables used. Also, there is no consensus on how to assess the relative importance the components of adaptive capacity. However, using the same set of fundamental adaptive capacity variable, genetic diversity, dispersal and migratory capacity and phenotypic plasticity^19^ in the same order of importance across the 17 lizard species increases the level of confidence of the vulnerability rankings of these species. This can guide prioritization of the species for conservation, although future studies will be crucial to evaluate the actual contributions of each variable to adaptive capacity across species in space and time.

Majority of our study lizards have high neutral genetic diversity (Supporting Information Table S5) and should this indeed be a good proxy for adaptive genetic variation, then it mean that they could adapt to climate change (Supporting Information Table S5). However, it is still debated if neutral genetic diversity is a good proxy for variation under natural selection^44^. Recent technological advances in next-generation sequencing show promise in identifying genetic markers for traits that confer thermal tolerance^45^. These markers could be useful to forecast adaptive responses of species to climate change in future studies^46^.

We ranked the dispersal capacity of lizards based on their body size, however, in general, lizards have limited dispersal capacity compared to large mammals and most birds. As yet, it is unclear if the localized mobility of lizards is sufficient to keep up with the pace of climate change^12,24,47^. Genetic characterization of the Cunningham’s skink^48^ and other lizard species^49–52^ showed population differentiation considerably above the F_ST_ level of 0.35, which is approximately the point at which the spread of advantageous alleles across a species’ range is prevented^53^. This suggests that the natural dispersal rates of these species are insufficient for them to track their climate niche even where habitats are contiguous and with no barriers to dispersal.

Moreover, the use of species distribution models to project changes in climatically suitable habitat has its own limitations that have been well documented elsewhere^54–56^. Species distribution models are sensitive to the choice of modelling algorithm^57–59^ and climate trajectory^60^. Therefore, different algorithms, and future changes in climate that do not conform to the climate trajectories used in the present study, could provide dramatically different exposure estimates and hence species vulnerability rankings. Other external stressors that were not accounted for, such as predation, competition and invasion, and potential disease outbreaks could substantially influence vulnerability to climate change^61^.

It has been shown that combining correlative and mechanistic SDMs improves the predictive accuracy of models^62^. Although we used only correlative SDM, future studies will benefit from the inclusion of mechanistic models in the projection of species distributions under climate change. Also, given that vulnerability categorization is relative, it is crucial to validate the vulnerability index, from even a small geographical area. Although this was beyond the scope of this study, it can be done via laboratory experiment or long-term field studies.

We have shown that the choice of CCVA frameworks and scoring schemes can influence the proportion and composition of species assigned to the different vulnerability categories. Sensitivity, exposure and adaptive capacity traits are unlikely to contribute equally to scores of species vulnerability to climate change^13,14^, and intuitively, weighted scores may provide a more realistic assessment. However, given the lack of empirical evidence to support vulnerability trait scores, it remains difficult to select an appropriate weighting scheme^3,5^. This is further complicated by the large number of variables that may be relevant for different taxonomic groups. Furthermore, even within a relatively small clade such as that investigated here, different variables may vary dramatically in their importance, across the species. Our study indicates that there is greater congruence between weighted and unweighted species’ vulnerability rankings when all three elements of vulnerability (exposure, sensitivity, and adaptive capacity) are integrated. Further studies are needed on other taxa, such as mammals and birds, and from various geographical areas to test the generality of our findings.

## Materials and Methods

### Study area and species

We assessed the vulnerability of lizards distributed along the Great Dividing Range (GDR) in southeastern Australia to climate change. The GDR traverses almost 3,500 km of the Australian continent, running from the west of Alps in southeast Victoria to north of Atherton in northeast Queensland^49^. The GDR is a key conservation area because it harbours globally endemic and endangered species^63^. As a result, it has been earmarked for the creation of a connectivity corridor to enhance species’ mobility and capacity to adapt to climate change^64^.

To demonstrate the generality and transferability of our framework, we selected lizards with a broad spectrum of natural history traits (e.g., body size, reproductive mode, thermal tolerance, habitat association) and for which data on life-history traits, ecology, genetics and occurrence records are readily available. We restricted our analysis to species whose entire distributional range fell within the boundary of the climate data used in this study (approx. 135.7–153.7 °E, 23.6–39.5 °S). Overall, 17 species, belonging to the families Scincidae (88%), Agamidae (6%) and Varanidae (6%), were analyzed.

### Assessing exposure

We used Maxent (version 3.3.3k)^65,66^ to model the current and future distribution of suitable habitat for the 17 lizard species. Maxent is one of the most frequently used correlative SDMs because of its high predictive performance, computational efficiency and ease of use (Elith *et al*. 2006; Phillips and Dudik, 2008; Phillips *et al*. 2009). We obtained species occurrence records from the Atlas of Living Australia (ALA; www.ala.org.au). We removed duplicate (points that occurred twice or more) and questionable records (i.e., records that had low positional accuracy and those fell outside the species’ known range or known records outside the range) and those that were collected before 1950. Overall, we included more than 31,000 unique occurrence points (i.e., one point per the spatial resolution considered) for the 17 species.

Current and future climate data, at a spatial resolution of 1 × 1 km, were derived from projections developed for the NSW and ACT Regional Climate Modelling (NARCliM) project^67^. Projections correspond to future climates simulated by four global climate models (GCMs): MIROC3.2-medres^68^, ECHAM5/MPI-OM^69^, CGCM3.1-T47^70^ and CSIRO-Mk3.0^71^, dynamically downscaled from 50 km resolution to 1 km and 250 m for south-eastern Australia using the Weather and Research Forecasting (WRF) Regional Climate Model^67^. Three alternate parameterizations of the WRF model (hereafter R1, R2, and R3), were used for downscaling, resulting in 12 future climate scenarios. The NARCliM project assumed the A2 emissions scenario^72^, which approximates the relative forcing and mean temperature trajectories of the RCP8.5 scenario^73^. In general, CGCM3.1 is a relatively hot/wet scenario, MIROC3.2 is a warm/wet scenario, CSIRO-Mk3.0 is a warm/dry scenario, while ECHAM5 projects major increases in temperature but little change in rainfall (hereafter: hot/similar precipitation scenario)^74^.

We use a suite of five predictor climatic variables that have been shown to predict the distribution of reptiles very well^13,14^. These included annual mean temperature, temperature seasonality, maximum temperature of the warmest month, minimum temperature of the coldest month and annual precipitation. Because microhabitats have been shown to play a significant role in thermal buffering and determining the presence of species at fine spatial resolutions^75,76^, and given that the most of the study species dwell in crevices of granite rock outcrops, we included an index of rock cover (Weathering Intensity Index)^77^ as a static predictor variable. We consider this to be an important addition because the presence of rock outcrops with suitable crevices will largely determine range filling (i.e., colonization of suitable habitats within the species range) under climate change.

We fitted the model using different combinations of Maxent features (i.e., linear, quadratic, product, threshold and hinge features), and varying levels of regularization, to control how tightly the model fitted the given occurrence points. The most realistic model settings as assessed by the smoothness of the response curves for our modelled species were linear, product, quadratic features, and a regularization multiplier of 1.5. To reduce over-prediction, background points were sampled from areas within 100 kilometres of occurrence localities. The fitted model was projected onto the current (20-year time period centred on 2000) and three future time slices (2030, 2050 and 2070). Future projections were constrained using a buffer of 300 km around the current distribution across all species to prevent the model predicting climatically suitable habitats in areas too far beyond the species’ range.

Model performance was evaluated using the area under the receiver operating characteristic curve (AUC) and the True Skill Statistic (TSS) based on 10-fold cross-validation. AUC scores range from 0 to 1, with values of 1 indicating perfect binary classifier accuracy and 0.5 suggesting that model performance is no better than random. TSS scores range from −1 to 1, where 1 indicates perfect agreement between test data and model predictions, and scores of 0 or less indicate performance no better than random^78^. Variables’ predictive ability and importance to the model were assessed by referring to their percentage contribution, and to the impacts of jack-knifing on model performance. A final model was fitted using all occurrence data, and habitat suitability maps were generated by projecting these models to current and future predictor data. The projected continuous habitat suitability maps were transformed into climatically suitable and unsuitable habitat using the equal training sensitivity and specificity logistic threshold^79,80^.

#### Analyses of model output

For each GCM, we computed the change in climatically suitable habitat between the current and future projections as the percentage change in the number of suitable grid cells. We also calculated the percentage of overlap between current and future suitable habitat and the percentage of suitable habitat lost or gained within protected areas. For the latter, we obtained a GIS layer of the Australian protected area network from the Collaborative Australian Protected Area Database (CAPAD 2014; available at www.environment.gov.au/parks/science/capad). Following Cabrelli and Hughes^13^, we assigned higher exposure scores to species projected to lose a higher proportion of their range and suitable habitat within protected areas, and to those with no overlap between current and future suitable habitat (Supporting Information Tables S1, S2 & S3).

### Assessing sensitivity

We undertook an intensive survey of the published literature to compile values for 11 traits that capture the sensitivity of each species to climate change. As described below, sensitivity was based on: narrow thermal tolerance^3,81^; geographically localised or restricted to a single climatic zone^15^; specialised habitat^10,15^ or dietary requirements^3^, slow reproductive rate^40^; temperature-dependent sex determination or small clutches^13^; low rate of offspring survival; and long generation length^7^ or life span^40^. Although some of these traits are correlated (e.g., generation length and life span), we included them in the analysis because these traits often interact to determine a species sensitivity to climate change. Unless otherwise stated, values for the above traits were obtained from Chapple^82^ and Greer^83^. We assessed each trait as follows (see also Supporting Information Table 4):

#### Physiological thermal tolerance

Changes in body temperature (T_b_) influences the physiological sensitivity and fitness of ectotherms. Very high Tb reduces an organisms’ fitness and can be lethal at the organism’s critical maximum temperature (CT_max_). An organism’s integrated fitness over some time is a function of its performance curve and the T_b_ it experiences. Therefore, the physiological impact of climate change will depend on an organism’s field T_b_ relative to its maximum performance temperature^84–86^. However, because some species may already occur in regions where environmental temperature is relatively close to their CT_max_^84–86^, we used the ratio of CT_max_ to the median temperature across the species’ range as a proxy for physiological thermal tolerance. The median temperature each species may be exposed to in the future was calculated by overlaying occurrence records with data describing future scenarios of mean annual temperature in southeastern Australia^67^. Species with a relatively lower ratio of CT_max_ to median temperature were given higher sensitivity scores than those with a higher ratio.

#### Range size

We estimated each species’ range size using the number of 100 × 100 km grid cells currently occupied. Species with relatively large range sizes were given lower sensitivity scores than those with smaller ranges.

#### Climatic zone

To obtain the number of climatic zones occupied by our study species, we overlaid their occurrence records with Koppen’s climate classification of Australia^83^. Species that occurred in multiple climatic zones were given lower sensitivity scores than those that occurred in only one zone.

#### Habitat requirements

Habitat generalists are more likely to adapt to changing conditions with climate change^87^. Indeed, species with more specialized habitats have been shown to respond negatively to climate change^3,88^. Consequently, habitat specialists were given higher sensitivity scores than generalists (those that occurred in multiple habitats).

#### Dietary requirements

Cabrelli and Hughes^13^ classified Australian skinks as specialists, borderline specialists and generalists based on the breadth of their prey types. We adopted this classification and gave diet specialists higher sensitivity scores than generalists.

#### Reproductive rate

Organisms that have fast reproductive rates respond less to climate change^88^. Thus, species that reproduce once or more within a year were given lower sensitivity scores than those that reproduce less frequently.

#### Reproductive mode

The nest temperature of many reptiles determines the sex of offspring (temperature-dependent sex determination, TSD). The correlation between temperature and offspring sex ratio implies that even modest increases in mean temperature may dramatically skew the sex ratio. High increases in temperature (>4 °C) could potentially eliminate production of male offspring thereby reducing population viability^89^. Although females may compensate for climatic variation via behavioral changes, such as nesting earlier in the season, digging deeper, or nesting in shade^90^, this may not compensate completely for climate change^91^. For this reason, species that lay eggs were given higher sensitivity scores than those that give birth to live-young.

#### Number of offspring

Organisms that have higher clutch sizes are more likely to produce more genetic variant individuals to trigger adaptation^87^, hence species that produce more offspring (≥5) per reproductive event were given a lower sensitivity score than those that have fewer offspring per event.

#### Offspring survival

Organisms with more offspring reaching sexual maturity are more likely to reproduce and have enough genetic variability to trigger adaptation^87^. For this reason, species with less than 50% of their offspring dying before reaching sexual maturity were given higher sensitivity scores than those with more than 50% of their offspring reaching sexual maturity.

#### Generation length

Species with longer generation times have slower life histories and lower reproductive output^87^. Consequently, species reaching sexual maturity within a long time (≥5 years) were given higher sensitivity scores than those with shorter juvenile stages.

#### Life span

Species that live longer are less susceptible to climate change because adaptation and range shifts occur over a long time^88^. Therefore, species that live for 10 years or more were given lower sensitivity scores than those with shorter lifespans.

### Assessing adaptive capacity

Although the theoretical basis of adaptive capacity is well understood, its quantification remains difficult and little information and guidance exists to inform its objective assessments^16,19^. We used four measures of adaptive capacity: genetic diversity; body size; habitat fragmentation; and microhabitat buffering (Supporting Information Table S5).

#### Genetic diversity

Given that evolutionary adaptation and plastic responses to climate change depend on the extent of genetic variation within species and among populations^32^, we used measures of genetic diversity as a proxy for adaptive potential. There are two type of genetic diversity: adaptive genetic diversity, which influences the fitness of individuals and populations and neutral genetic diversity, which confers no advantage, but may provide proxy for adaptive variation and means for monitoring gene flow and other demographic processes^53^. Recent genomics analyses suggest that most adaptive genetic responses are based on small effect in many genes, rather than a few genes of large effect^92^. These considerations suggest that evolutionary potential (a key component of adaptive capacity) is better predicted by overall genetic diversity than a focus on individual genes^93^. Here we used neutral genetic variation (as measured by expected heterozygosity) as a proxy for adaptive potential. Expected heterozygosity (He) for the lizards (measured by microsatellite markers) were obtained from the published literature (for references see Supporting Information Table S5). In principle, species with relatively high genetic diversity are expected to have greater adaptive potential, hence we assigned higher adaptive capacity scores to species with mean He > 0.8.

#### Dispersal capacity

Empirical data on the dispersal of most lizards is rare. However, given their strong correlation, we used body size as a surrogate for dispersal capacity^94^. In general, the larger the organism, the more mobile it is, hence larger lizards (measured by snout-vent length, SVL) were assigned relatively higher dispersal capacity, and thus adaptive capacity, than smaller ones.

#### Habitat loss and fragmentation

The availability of suitable habitat and degree of connectivity of the landscape can impede or enhance movement of organisms, thereby influencing their response to climate change^15,95,96^. To assess the availability of suitable habitat ant degree of habitat fragmentation for individual species, we overlaid species’ current range maps with a GIS layer of the land use/land cover of Australia (Australian Land Use and Management [ALUM] Classification, version 7, May 2010, available at www.abs.gov.au). For each species, we calculated the percentage of occupied grid cells that fell within pasture, crop and modified lands. We assigned a higher adaptive capacity score to species with more than 40% of their occupied grid cells within pasture, crop and modified lands, and a lower score to those with less than 10% of the occupied grid cells within these land cover types.

#### Microhabitat buffering

The use of microhabitat features that moderate temperature and extreme weather conditions can influence the capacity of species to cope with climate change^75,76^. Hence, species that used rock crevices and tree hollows as retreat sites were given higher adaptive capacity scores than those that used leaf litter or bare ground.

### Vulnerability framework and scoring

We scored the variables of the three elements of vulnerability on an ordinal scale using two scoring schemes: unweighted and weighted (Table 1). In the unweighted scoring scheme, we considered the variables within each element as equally important and were awarded a maximum score of three points and a minimum score of one point following Gardali, *et al*.^16^. In the weighted scheme, the variables within each element were ranked according to their perceived relative contribution to vulnerability as deemed by a panel of experts^13,14^. Higher maximum scores were awarded to the variables that contributed more to climate vulnerability, with categories within variables awarded a minimum score of one and a median score of half the maximum score following Cabrelli, *et al*.^14^. For example, of the 11 sensitivity traits used in this study, thermal tolerance was regarded the most important trait and so it was awarded the highest maximum score of 11 for species with narrow thermal tolerance, a score of 5.5 for species with moderate tolerance, and a score of one for species with wide tolerance. Life span was regarded the least important of the traits and was therefore awarded the lowest maximum score of three for short-lived species, a score of two for species with moderate life-spans, and a score of one for long-lived species (Table 1).

**Table 1:** Description of the variable categories and their scores for the three elements of climate change vulnerability as applied to assess the vulnerability of 17 lizards along the Great Dividing Range of Australia (for references see Supporting Information Tables S4 and S5). For geographic range size, N refers to the number of 100 × 100 km grid cells occupied by the species.

**Variable**	**Category (description)**	**Unweighted score**	**Weighted score**
**EXPOSURE**			
Change in area of current climatically suitable habitat	Increase or little change (i.e. <10% decrease)	1	1
	10–50% decrease	2	2
	>50% decrease	3	4
Overlap between current and future suitable habitat	>50%	1	1
	10–50%	2	2.5
	<10%	3	5
Suitable habitat within protected areas	>50%	1	1
	10–50%	2	1.5
	<10%	3	3
**SENSITIVITY**			
Physiological tolerance	Wide (CT_max_:median temp >3 °C)	1	1
	Moderate (CT_max_:median temp = 1.5–3 °C)	2	5.5
	Narrow (CT_max_:median temp <1.5 °C)	3	11
Geographic range size	Large (N ≥ 50)	1	1
	Moderate (25 ≤ N < 50)	2	5
	Small (N < 25)	3	10
Climatic zones	Multiregional (>3 climate regions)	1	1
	Moderate (2–3 climate regions)	2	4.5
	Narrow (1 climate region)	3	9
Habitat requirement	Generalist (>3 habitat types)	1	1
	Moderate (2–3 habitat types)	2	4
	Specialist (1 habitat type only)	3	8
Dietary requirement	Generalists (omnivore, or exploits a wide variety of food)	1	1
	Moderate (able to tolerate some variety of food)	2	3.5
	Specialist (restricted to a particular food item)	3	7
Reproduction rate	Perennial	1	1
	Annual	2	3
	Biennial	3	6
Reproduction mode	Viviparous	1	1
	Ovoviviparous	2	3
	Oviparous/temperature dependent sex	3	6
Mean Clutch size	Large (≥5)	1	1
	Moderate ( = 3–4)	2	2.5
	Small (2)	3	5
Offspring survival rate	>80% of offspring reach sexual maturity	1	1
	50–80% of offspring reach sexual maturity	2	2.5
	<50% of offspring reach sexual maturity	3	5
Generation length	Short (≤2 years)	1	1
	Moderate (3–4 years)	2	2
	Long (≥5 years)	3	4
Life span	Long-lived (>10 years)	1	1
	Moderate (5–10 years)	2	2
	Short-lived (<5 years)	3	3
**ADAPTIVE CAPACITY**			
Dispersal capacity	Low (SVL < 100 mm)	1	1
	Moderate (100 mm < SVL < 200 mm)	2	3
	high (SVL > 200 mm)	3	6
Genetic variability and evolutionary potential	Low (He > 0.6)	1	1
	Moderate (0.6 < He < 0.8)	2	2.5
	high (He > 0.8)	3	5
Habitat fragmentation or barriers to dispersal	high (>50% of range within pasture, crop and modified lands [PCMLs])	1	1
	Moderate (10–50% of range within PCMLs)	2	2
	Low (<10% of range within PCMLs)	3	4
Microhabitat buffer	Uses open ground only	1	1
	Uses ground litter cover and tree bark	2	2
	Uses deep rock crevices, burrows, under rocks and holes in logs	3	3

Although these scoring schemes are highly arbitrary, they provide an easy-to-use approach to convert continuous variables into ordinal (high or low) categorization. This approach has been used in similar assessments^3,16,87,97^.

### Climate vulnerability score and ranking threshold

To generate a climate change vulnerability score, we first divided the sum of the scores for each of the three elements of vulnerability by their respective potential maximum score to generate exposure score (*Es*), sensitivity score (*Ss*) and adaptive capacity score (*As*). We then computed vulnerability score with adaptive capacity (ESA) as (*Es* + *Ss*) − *As* and without adaptive capacity (ES) as *Es* + *Ss*. Computing ESA as (*Es* × *Ss*)/*As* did not change the outcome of the vulnerability rankings. We applied two of the commonly used vulnerability ranking thresholds. Firstly, following Cabrelli, *et al*.^14^, species with ESA/ES ≥67 were ranked as high vulnerability, those with ESA/ES from 33 to 66 were ranked as moderate vulnerability and those with ESA/ES < 33 were classified as low vulnerability. Secondly, following Dawson, *et al*.^10^ and Foden, *et al*.^3^, we assigned species to four vulnerability categories: high, potential adapters, potential persisters and high latent risk. High vulnerability species are those that have high sensitivity, high exposure and low adaptive capacity to climate change. Potential adapters are the species that are highly exposed and sensitive to climate change, but have high capacity to adapt and tolerate the impacts of the change (Dawson *et al*., 2011; Foden *et al*., 2013). Potential persisters are species that are highly exposed and have low adaptive capacity, but are not sensitive to climate change, while species with high latent risk are those with low exposure, but high sensitivity and low adaptive capacity to climate change (Dawson *et al*., 2011; Foden *et al*., 2013).

In the first classification, vulnerability categorization based on the raw continuous values produced similar results as categorization based on ranked values for exposure, sensitivity and adaptive capacity. The second classification however required that we first rank the species as either high, moderate or low exposure, sensitivity and adaptive capacity before determining their vulnerability status. We evaluated the relationship between the two assessment methods (i.e., with and without considering adaptive capacity) and scoring schemes (i.e., unweighted and weighted) using the Spearman’s rank correlation coefficient and analysed the difference between them using the Fisher’s exact test.

## Electronic supplementary material

Supplementary Information
