# Supplementary material for: Influence of adaptive capacity on the outcome of climate change vulnerability assessment
Source: Sci Rep. 2017 Oct 11;7:12979. doi: 10.1038/s41598-017-13245-y (PMC5636830; doi:10.1038/s41598-017-13245-y)
Supplement: Supplementary file 1 — Supplementary Information [file 41598_2017_13245_MOESM1_ESM.pdf]

Influence of adaptive capacity on the outcome of climate change vulnerability assessment

Benjamin Y. Ofori[1,2,*], Adam J. Stow[1], John B. Baumgartner[1] & Linda J. Beaumont[1]

[1]Department of Biological Sciences, Macquarie University, North Ryde, Macquarie Park, NSW 2019, Australia

[2]Department of Animal Biology and Conservation Science, University of Ghana, Legon-Accra, Ghana

[*]Corresponding author; E-mail: byofori@yahoo.com

Table S1: Percentage change in the area of climatically suitable habitat for 17 Australian lizard species under four climate scenarios, for 2030, 2050 and 2070 and time horizons (reference year is 2000). Habitat suitability was modelled at 1 × 1 km resolution using Maxent.

	**Hot/wet**			**Warm/dry**			**Hot/similar precipitation**			**Warm/wet**		
**Species**	**2030**	**2050**	**2070**	**2030**	**2050**	**2070**	**2030**	**2050**	**2070**	**2030**	**2050**	**2070**
Amphibolurus muricatus	−20.0	−31.6	−41.8	−24.3	−35.3	−45.5	−39.9	−38.9	−39.5	−13.5	−21.1	−28.6
Ctenotus taeniolatus	−23.4	−28.1	−32.2	−12.1	−13.6	−15.1	−45.6	−28.3	−8.0	−8.3	−2.2	−11.5
Cyclodomorphus gerrardii	−23.7	−34.4	−42.3	−27.9	−37.4	−47.8	−32.9	−30.5	−26.5	1.4	13.0	26.2
Egernia cunninghami	−30.7	−41.4	−47.4	−36.7	−44.9	−50.8	−63.3	−46.2	−22.5	−29.5	−30.0	−27.2
Egernia frerei	−38.4	−52.6	−64.5	−45.6	−61.6	−73.4	−46.0	−48.4	−51.3	−0.9	11.1	16.8
Egernia kingii	−32.9	−43.8	−52.5	−29.7	−45.7	−58.0	−33.4	−34.8	−35.6	−0.4	3.4	8.1
Egernia stokesii	−1.5	11.5	61.7	−21.4	−28.8	−35.6	−46.9	−17.4	149.7	−13.2	−8.3	74.0
Egernia striolata	18.0	4.1	−9.7	−29.7	−19.3	11.1	45.6	27.0	−24.3	12.0	−12.4	−28.7
Egernia whitii	−6.1	11.9	34.4	11.5	26.7	43.6	−12.4	14.4	50.0	11.3	44.3	88.5
Eulamprus heatwolei	−33.6	−50.0	−64.9	−29.3	−46.2	−61.2	−52.6	−57.5	−63.1	−21.2	−31.5	−42.6
Eulamprus kosciuskoi	−54.5	−71.4	−82.7	−48.0	−65.6	−77.0	−76.2	−79.6	−82.6	−38.1	−50.2	−60.7
Eulamprus leuraensis	−70.6	−97.0	−99.9	−62.1	−97.3	−99.9	−90.4	−99.9	−100.0	−27.2	−63.8	−79.5
Eulamprus quoyii	−21.3	−27.6	−32.6	−17.8	−24.5	−29.9	−32.1	−30.4	−28.3	−12.4	−16.2	−20.0
Eulamprus tympanum	−34.4	−56.8	−74.8	−29.1	−49.5	−67.1	−49.5	−62.2	−74.0	−21.0	−36.7	−51.4
Lampropholis delicata	−23.1	−37.4	−50.1	−30.7	−44.9	−58.2	−44.5	−42.6	−42.1	−12.3	−18.5	−25.5
Lissolepis coventryi	25.6	66.2	106.9	27.5	45.0	58.7	25.5	79.1	135.9	37.2	76.6	119.6
Varanus varius	19.0	30.1	51.0	−7.3	−4.8	−2.1	−2.8	11.1	32.2	29.6	78.9	127.8

Table S2: Percentage of future climatically suitable habitat that overlaps with current suitable habitat for 17 Australian lizard species under four contrasting but equally plausible climate scenarios (hot/wet; warm/dry; hot/similar precipitation; and warm/wet) at 2030, 2050 and 2070. Habitat suitability was modelled at 1 × 1 km resolution using Maxent.

	**Hot/wet**			**Warm/dry**			**Hot/similar precipitation**			**Warm/wet**		
**Species/ Time**	**2030**	**2050**	**2070**	**2030**	**2050**	**2070**	**2030**	**2050**	**2070**	**2030**	**2050**	**2070**
Amphibolurus muricatus	79.9	68.4	58.0	75.6	64.6	54.3	60.1	60.9	60.3	86.3	78.6	71.0
Ctenotus taeniolatus	76.5	71.0	63.4	84.8	81.0	75.9	54.4	71.2	83.1	85.6	89.8	88.0
Cyclodomorphus gerrardii	75.9	65.5	57.4	71.9	62.2	51.5	67.1	69.3	71.3	94.9	92.6	87.3
Egernia cunninghami	68.3	57.6	48.2	62.4	54.1	47.4	36.6	52.3	67.8	68.9	68.1	68.1
Egernia frerei	60.6	47.3	35.5	54.4	38.4	26.6	54.0	51.6	48.7	91.3	95.8	92.2
Egernia kingii	67.1	56.2	45.7	70.2	54.3	42.0	66.5	65.2	64.1	92.5	94.5	93.9
Egernia stokesii	87.1	87.2	85.6	75.4	66.0	57.6	51.1	65.3	85.4	81.6	80.9	81.5
Egernia striolata	94.2	88.8	77.4	68.6	75.0	83.2	99.9	83.6	62.5	98.3	80.0	64.0
Egernia whitii	91.2	93.7	93.7	96.3	95.8	95.1	85.0	92.2	93.7	96.5	96.6	96.3
Eulamprus heatwolei	66.3	49.7	34.7	70.3	53.3	38.2	47.4	42.2	36.5	78.0	67.7	56.3
Eulamprus kosciuskoi	45.5	28.6	17.3	25.0	34.4	23.0	23.8	20.4	17.4	61.9	49.8	39.3
Eulamprus leuraensis	30.0	29.8	0.0	37.0	2.6	0.1	9.6	0.1	0.0	63.6	25.4	6.3
Eulamprus quoyii	78.7	72.0	67.4	81.8	75.5	70.1	67.9	69.6	71.7	87.0	83.5	80.0
Eulamprus tympanum	65.6	43.2	25.2	70.8	50.5	32.9	50.5	37.8	26.0	78.5	62.8	47.7
Lampropholis delicata	76.7	62.4	49.7	69.3	55.0	41.6	55.4	57.2	57.7	87.3	81.2	74.1
Lissolepis coventryi	98.9	99.1	98.7	99.5	98.5	97.4	98.9	99.7	99.9	99.1	98.7	98.1
Varanus varius	95.8	92.3	87.2	85.5	81.6	77.0	90.9	89.4	86.2	99.9	98.4	94.9

Table S3: Percentage change in climatically suitable habitat within Protected Areas for 17 Australian lizard species under four contrasting but equally plausible climate scenarios (hot/wet; warm/dry; hot/similar precipitation; and warm/wet) at 2030, 2050 and 2070 (reference year is 2000). Habitat suitability was modelled at 1 × 1 km resolution using Maxent.

	**Hot/wet**			**Warm/dry**			**Hot/similar precipitation**			**Warm/wet**		
**Species**	**2030**	**2050**	**2070**	**2030**	**2050**	**2070**	**2030**	**2050**	**2070**	**2030**	**2050**	**2070**
Amphibolurus muricatus	−7.9	−16.1	−25.2	−10.5	−18.4	−26.7	−19.4	−20.2	−22.6	−5.1	−84.9	−12.5
Ctenotus taeniolatus	−19.5	−21.7	−24.4	−6.9	−4.4	−3.1	−43.5	−21.5	−1.8	−6.4	−6.0	−15.0
Cyclodomorphus gerrardii	−21.4	−27.7	−35.9	−20.9	−29.9	−40.5	−32.5	−27.1	−20.7	−0.4	15.9	33.6
Egernia cunninghami	−25.0	−29.1	−33.2	−22.9	−27.1	−32.9	−54.7	−29.2	−9.0	−20.9	−18.6	−16.0
Egernia frerei	−30.5	−43.9	−56.7	−36.5	−54.2	−68.3	−39.1	−42.2	−45.6	0.5	7.1	9.3
Egernia kingii	−32.2	−41.7	−49.2	−24.2	−38.2	−50.7	−34.3	−33.8	−33.1	−7.0	−2.8	2.4
Egernia stokesii	−0.8	−0.4	100.0	−3.5	−3.8	−3.2	−44.8	−21.9	299.7	−21.0	−2.8	−284.8
Egernia striolata	38.9	30.8	23.3	−31.7	−22.1	43.4	69.3	38.7	15.6	18.3	6.4	−3.5
Egernia whitii	−13.7	−7.3	1.7	−2.3	4.7	13.6	−25.4	−10.4	9.7	1.4	19.8	42.3
Eulamprus heatwolei	−28.0	−41.5	−54.7	−22.5	−36.7	−50.6	−45.5	−48.4	−53.1	−16.8	−24.5	−33.8
Eulamprus kosciuskoi	−43.4	−60.4	−74.0	−36.9	−53.8	−66.6	−70.5	−72.9	−75.6	−29.0	−39.4	−49.0
Eulamprus leuraensis	−60.6	−94.5	−99.9	−59.0	−95.2	−99.8	−88.1	−99.8	−100.0	−35.6	−67.0	−73.8
Eulamprus quoyii	−10.6	−16.6	−21.3	−10.3	−15.2	−18.9	−18.9	−17.9	−17.4	−7.7	−10.2	−12.6
Eulamprus tympanum	−26.1	−43.3	−59.7	−22.6	−38.8	−53.2	−45.8	−53.5	−61.8	−12.2	−21.5	−31.3
Lampropholis delicata	−12.3	−22.3	−34.0	−18.2	−29.4	−42.4	−34.1	−30.0	−28.5	−6.8	−8.4	−12.4
Lissolepis coventryi	20.7	43.1	68.1	18.4	28.5	35.4	17.9	48.1	81.1	26.5	50.9	82.1
Varanus varius	13.6	28.4	43.0	−0.4	−10.1	−19.4	0.7	20.7	39.3	21.2	−50.3	−73.2

Table S4: Sensitivity traits for 17 Australian lizard species. CT_{max}/median temp represents physiological thermal tolerance, calculated as the ratio of critical maximum temperature (CT_{max}) to the median temperature across the species' range. Geographic range size is the number (N) of 100 × 100 km grid cells that contain occurrence records: Large is $N \geq 50$; Moderate is $25 \leq N < 50$; Small is $N < 25$. Habitat requirements: Specialists are species that are restricted to only one habitat type; Generalists occur in more than three habitat types. Dietary requirements: Specialists are species that are restricted to a particular food item; Generalists exploit a wide variety of food items. Climate zone: Multiregional species occur within more than three climate regions; Moderate occur within two to three climate regions; Narrow occur in only one climate region.

**SPECIES**	**Ctmax/median temp**	**Geographic range size**	**Habitat requirement**	**Dietary requirement**	**Climatic zone**	**Reproduction rate**	**Reproduction mode**
Amphibolurus muricatus	1.54	Large (99)	Specialist	Generalist	Multiregion	Annual	Oviparous
Ctenotus taeniolatus	1.40	Moderate (42)	Specialist	Generalist	Multiregion	Annual	Viviparous
Cyclodomorphus gerrardii	1.51	Large (53)	Generalist	Specialist	Moderate	Annual	Viviparous
Egernia cunninghami	1.53	Large (50)	Generalist	Generalist	Moderate	Annual	Viviparous
Egernia frerei	1.40	Moderate (26)	Specialist	Generalist	Multiregion	Annual	Viviparous
Egernia kingii	1.22	Large (152)	Generalist	Generalist	Multiregion	Annual	Viviparous
Egernia stokesii	1.15	Moderate (36)	Generalist	Generalist	Multiregion	Annual	Viviparous
Egernia striolata	1.34	Large 167)	Generalist	Generalist	Multiregion	Annual	Viviparous
Egernia whitii	1.60	Small (20)	Generalist	Generalist	Multiregion	Annual	Viviparous
Eulamprus heatwolei	1.58	Moderate (43)	Generalist	Generalist	Multiregion	Annual	Viviparous
Eulamprus kosciuskoi	1.78	Small (8)	Specialist	Generalist	Moderate	Annual	Viviparous
Eulamprus leuraensis	1.62	Small (2)	Specialist	Generalist	very Narrow	Annual	Viviparous
Eulamprus quoyii	1.44	Large (250)	Specialist	Generalist	Multiregion	Biannual	Viviparous
Eulamprus tympanum	1.62	Small (22)	Generalist	Generalist	Moderate	Annual	Viviparous
Lampropholis delicata	1.39	Large (127)	Generalist	Generalist	Multiregion	Annual	Viviparous

Lissolepis coventryi	1.58	Small (13)	Specialist	Generalist	Narrow	Annual	Viviparous
Varanus varius	1.52	Large (53)	Generalist	Generalist	Multiregion	Annual	Oviparous

Table S4: Continued

Species	Mean Clutch size	Offspring survival	Maturation length (years)	Life span (years)	References
Amphibolurus muricatus	6	<50%	1 to 2	> 10	1, 2
Ctenotus taeniolatus	10	<50%	1 to 2	4 to 6	1, 3, 4
Cyclodomorphus gerrardii	20	<50%	2	> 10	1, 3, 4
Egernia cunninghami	6	<50%	5	> 10	1, 3–8
Egernia frerei	7	<50%	4	> 10	3, 4, 9
Egernia kingii	6	<50%	3	> 10	1, 3, 4, 10
Egernia stokesii	5	<50%	5	> 10	1–4
Egernia striolata	3	<50%	2 to 3	8 to 10	1, 3, 4
Egernia whitii	3	<50%	2 to 3	>8.5	1, 3, 4
Eulamprus heatwolei	4	<50%	2 to 3	> 10	1, 3, 4
Eulamprus kosciuskoi	3	<50%	2 to 3	> 10	1, 3, 4
Eulamprus leuraensis	3	<50%	3	> 10	1, 3, 4, 9, 11
Eulamprus quoyii	4	<50%	2 to 3	> 10	1, 3, 4, 9
Eulamprus tympanum	3	<50%	2 to 3	> 10	1, 3, 4, 9, 12
Lampropholis delicata	4	<50%	2	8 to 10	1, 4
Lissolepis coventryi	3	<50%	2	> 8	1, 3, 4, 13
Varanus varius	9	<50%	3 to 4	> 10	1, 2

Table S5: Adaptive capacity traits for 17 Australian lizard species sourced from the published literature. Body size was used as proxy for dispersal capacity. In general, species with relatively large body size are presumed to have higher dispersal rate. Also, species with higher neutral genetic diversity are presumed to have relatively higher adaptive potential.

**SPECIES**	**Body size (SVL in mm)**	**Genetic diversity (mean He)**	**Habitat fragmentation**	**Microhabitat buffer**	**References**
Amphibolurus muricatus	39–125	0.856 (0.232–0.964)	>50%	OR, OL	1, 14–18
Ctenotus taeniolatus	80	0.8138 (0.649–0.887)	>50%	UL, UR	14, 19, 20, 21
Cyclodomorphus gerrardii	255	0.817 (0.54–0.95)	>50%	UL, UR	19, 22
Egernia cunninghami	230–250	0.862 (0.516–0.955)	>50%	UR, UL	6, 7, 14, 23
Egernia frerei	180–200	0.66	10–50%	UL	3, 19, 24
Egernia kingii	200–230	0.798	>50%	UR, BW	3, 19
Egernia stokesii	155–190	0.68 (0.1–0.9)	>50%	UR, BW, TH	25, 26
Egernia striolata	100–199	0.875 (0.763–0.915)	>50%	UR, UL	19, 27, 28
Egernia whitii	75–110	0.9145 (0.874–0.933)	>50%	UR	3, 14, 19, 29, 30
Eulamprus heatwolei	80	0.68 (0.314–0.909)	>50%	UR	19, 23, 31
Eulamprus kosciuskoi	80	0.643 (0.271–0.898)	>50%	UR, BW	19, 32
Eulamprus leuraensis	80–85	0.7 (0.32–0.81)	>50%	DGT	19, 33
Eulamprus quoyii	90–118	0.674	10–50%	UR, UL	14, 19, 34
Eulamprus tympanum	80–96	0.674	>50%	UR, UL	19, 23, 35
Lampropholis delicata	34–46	0.701 (0.114–0.959)	>50%	LL, UB, UR	14, 19, 36
Lissolepis coventryi	100–130	0.668 (0.216–0.817)	>50%	UL, BW, UR	1, 13
Varanus varius	200–2000	0.934 (0.882–0.963)	>50%	LL, OR	14, 37, 38

Table S6: Unweighted (I) and weighted (II) vulnerability scores for 17 Australian lizards under projected climate change by 2030, 2050 and 2070. Vulnerability was assessed using four contrasting future climate scenarios that are equally plausible: hot/wet; warm/dry; hot/similar precipitation; and warm/wet. Analysis was performed using all three elements of vulnerability (ESA; exposure, sensitivity and adaptive capacity) and using exposure and sensitivity only (ES). Score > 0.67 is high, from 0.33 to 0.67 is moderate and < 0.33 is low.

**Hot/wet**	**2030**		**2050**		**2070**		**2030**		**2050**		**2070**	
**Species**	**ES I**	**ESA I**	**ES I**	**ESA I**	**ES I**	**ESA I**	**ES II**	**ESA II**	**ES II**	**ESA II**	**ES II**	**ESA II**
Amphibolurus muricatus	0.51	0.48	0.56	0.52	0.57	0.52	0.42	0.44	0.46	0.47	0.46	0.47
Ctenotus taeniolatus	0.58	0.5	0.58	0.50	0.58	0.50	0.50	0.48	0.50	0.48	0.50	0.48
Cyclodomorphus gerrardii	0.55	0.42	0.55	0.42	0.55	0.42	0.43	0.34	0.43	0.34	0.43	0.34
Egernia cunninghami	0.55	0.42	0.55	0.42	0.61	0.46	0.41	0.33	0.41	0.33	0.45	0.36
Egernia frerei	0.58	0.47	0.69	0.54	0.75	0.58	0.50	0.47	0.64	0.57	0.66	0.58
Egernia kingii	0.55	0.45	0.55	0.45	0.66	0.52	0.44	0.40	0.44	0.40	0.57	0.48
Egernia stokesii	0.51	0.45	0.45	0.41	0.51	0.45	0.41	0.43	0.37	0.40	0.41	0.43
Egernia striolata	0.49	0.41	0.44	0.38	0.44	0.38	0.38	0.36	0.34	0.34	0.34	0.34
Egernia whitii	0.57	0.49	0.45	0.41	0.45	0.41	0.45	0.45	0.36	0.39	0.36	0.39
Eulamprus heatwolei	0.55	0.51	0.66	0.58	0.72	0.62	0.41	0.47	0.56	0.57	0.58	0.58
Eulamprus kosciuskoi	0.72	0.62	0.78	0.66	0.78	0.66	0.67	0.64	0.71	0.67	0.69	0.66
Eulamprus leuraensis	0.79	0.67	0.79	0.67	0.79	0.67	0.74	0.69	0.74	0.69	0.72	0.68
Eulamprus quoyii	0.58	0.5	0.58	0.50	0.58	0.50	0.48	0.50	0.48	0.50	0.48	0.50
Eulamprus tympanum	0.58	0.53	0.69	0.60	0.75	0.64	0.47	0.51	0.62	0.60	0.64	0.62
Lampropholis delicata	0.57	0.52	0.57	0.52	0.62	0.55	0.43	0.48	0.43	0.48	0.51	0.54
Lissolepis coventryi	0.53	0.46	0.53	0.46	0.53	0.46	0.48	0.48	0.48	0.48	0.48	0.48
Varanus varius	0.47	0.4	0.47	0.40	0.47	0.40	0.35	0.29	0.35	0.29	0.35	0.29

Table S6: Continued

Warm/dry	2030		2050		2070		2030		2050		2070	
**Species**	**ES I**	**ESA I**	**ES I**	**ESA I**	**ES I**	**ESA I**	**ES II**	**ESA II**	**ES II**	**ESA II**	**ES II**	**ESA II**
Amphibolurus muricatus	0.57	0.52	0.57	0.52	0.57	0.52	0.46	0.47	0.46	0.47	0.46	0.47
Ctenotus taeniolatus	0.53	0.46	0.53	0.46	0.53	0.46	0.46	0.45	0.46	0.45	0.46	0.45
Cyclodomorphus gerrardii	0.55	0.42	0.55	0.42	0.55	0.42	0.43	0.34	0.43	0.34	0.43	0.34
Egernia cunninghami	0.55	0.42	0.55	0.42	0.66	0.50	0.41	0.33	0.41	0.33	0.55	0.42
Egernia frerei	0.58	0.47	0.64	0.51	0.75	0.58	0.50	0.47	0.56	0.51	0.69	0.60
Egernia kingii	0.55	0.45	0.55	0.45	0.72	0.56	0.44	0.40	0.44	0.40	0.63	0.52
Egernia stokesii	0.51	0.45	0.51	0.45	0.51	0.45	0.41	0.43	0.41	0.43	0.41	0.43
Egernia striolata	0.49	0.41	0.55	0.45	0.44	0.38	0.38	0.36	0.42	0.39	0.34	0.34
Egernia whitii	0.45	0.41	0.45	0.41	0.45	0.41	0.36	0.39	0.36	0.39	0.36	0.39
Eulamprus heatwolei	0.55	0.51	0.55	0.51	0.72	0.62	0.41	0.47	0.41	0.47	0.60	0.59
Eulamprus kosciuskoi	0.67	0.58	0.78	0.66	0.78	0.66	0.59	0.59	0.71	0.67	0.71	0.67
Eulamprus leuraensis	0.79	0.67	0.85	0.70	0.85	0.70	0.74	0.69	0.85	0.76	0.85	0.76
Eulamprus quoyii	0.58	0.50	0.58	0.50	0.58	0.50	0.48	0.50	0.48	0.50	0.48	0.50
Eulamprus tympanum	0.58	0.53	0.58	0.53	0.75	0.64	0.47	0.51	0.47	0.51	0.62	0.60
Lampropholis delicata	0.57	0.52	0.57	0.52	0.68	0.59	0.43	0.48	0.43	0.48	0.53	0.55
Lissolepis coventryi	0.59	0.50	0.53	0.46	0.53	0.46	0.52	0.51	0.48	0.48	0.48	0.48
Varanus varius	0.47	0.40	0.53	0.43	0.53	0.43	0.35	0.29	0.39	0.32	0.39	0.32

Table S6: Continued

Hot/similar precipitation	2030		2050		2070		2030		2050		2070	
**Species**	**ES I**	**ESA I**	**ES I**	**ESA I**	**ES I**	**ESA I**	**ES II**	**ESA II**	**ES II**	**ESA II**	**ES II**	**ESA II**
Amphibolurus muricatus	0.57	0.52	0.57	0.52	0.57	0.52	0.46	0.47	0.46	0.47	0.46	0.47
Ctenotus taeniolatus	0.58	0.50	0.58	0.50	0.47	0.42	0.50	0.48	0.50	0.48	0.41	0.42
Cyclodomorphus gerrardii	0.55	0.42	0.55	0.42	0.55	0.42	0.43	0.34	0.43	0.34	0.43	0.34
Egernia cunninghami	0.72	0.53	0.55	0.42	0.49	0.39	0.60	0.45	0.41	0.33	0.37	0.30
Egernia frerei	0.58	0.47	0.58	0.47	0.69	0.54	0.50	0.47	0.50	0.47	0.64	0.57
Egernia kingii	0.55	0.45	0.55	0.45	0.55	0.45	0.44	0.40	0.44	0.40	0.44	0.40
Egernia stokesii	0.57	0.49	0.57	0.49	0.45	0.41	0.45	0.46	0.45	0.46	0.37	0.40
Egernia striolata	0.44	0.38	0.44	0.38	0.49	0.41	0.34	0.34	0.34	0.34	0.38	0.36
Egernia whitii	0.57	0.49	0.51	0.45	0.45	0.41	0.45	0.45	0.41	0.42	0.36	0.39
Eulamprus heatwolei	0.66	0.58	0.66	0.58	0.72	0.62	0.56	0.57	0.56	0.57	0.60	0.59
Eulamprus kosciuskoi	0.78	0.66	0.78	0.66	0.78	0.66	0.71	0.67	0.71	0.67	0.71	0.67
Eulamprus leuraensis	0.85	0.70	0.85	0.70	0.85	0.70	0.85	0.76	0.85	0.76	0.85	0.76
Eulamprus quoyii	0.58	0.50	0.58	0.50	0.58	0.50	0.48	0.50	0.48	0.50	0.48	0.50
Eulamprus tympanum	0.58	0.53	0.75	0.64	0.75	0.64	0.47	0.51	0.66	0.63	0.66	0.63
Lampropholis delicata	0.57	0.52	0.57	0.52	0.57	0.52	0.43	0.48	0.43	0.48	0.43	0.48
Lissolepis coventryi	0.53	0.46	0.53	0.46	0.53	0.46	0.48	0.48	0.48	0.48	0.48	0.48
Varanus varius	0.47	0.40	0.47	0.40	0.47	0.40	0.35	0.29	0.35	0.29	0.35	0.29

Table S6: Continued

Warm/wet	2030		2050		2070		2030		2050		2070	
**Species**	**ES I**	**ESA I**	**ES I**	**ESA I**	**ES I**	**ESA I**	**ES II**	**ESA II**	**ES II**	**ESA II**	**ES II**	**ESA II**
Amphibolurus muricatus	0.51	0.48	0.62	0.55	0.57	0.52	0.42	0.44	0.50	0.50	0.46	0.47
Ctenotus taeniolatus	0.47	0.42	0.47	0.42	0.57	0.49	0.41	0.42	0.41	0.42	0.50	0.48
Cyclodomorphus gerrardii	0.44	0.35	0.44	0.35	0.45	0.36	0.35	0.29	0.35	0.29	0.35	0.29
Egernia cunninghami	0.55	0.42	0.55	0.42	0.55	0.42	0.41	0.33	0.41	0.33	0.41	0.33
Egernia frerei	0.47	0.40	0.47	0.40	0.48	0.41	0.41	0.42	0.41	0.42	0.41	0.42
Egernia kingii	0.44	0.38	0.44	0.38	0.44	0.38	0.36	0.34	0.36	0.34	0.36	0.34
Egernia stokesii	0.57	0.49	0.45	0.41	0.44	0.40	0.45	0.46	0.37	0.40	0.37	0.40
Egernia striolata	0.44	0.38	0.49	0.41	0.49	0.41	0.34	0.34	0.38	0.36	0.38	0.36
Egernia whitii	0.45	0.41	0.45	0.41	0.42	0.39	0.36	0.39	0.36	0.39	0.36	0.39
Eulamprus heatwolei	0.55	0.51	0.55	0.51	0.54	0.50	0.41	0.47	0.41	0.47	0.41	0.47
Eulamprus kosciuskoi	0.61	0.55	0.72	0.62	0.72	0.62	0.53	0.55	0.67	0.64	0.67	0.64
Eulamprus leuraensis	0.63	0.56	0.79	0.67	0.85	0.70	0.56	0.57	0.74	0.69	0.85	0.76
Eulamprus quoyii	0.53	0.46	0.58	0.50	0.58	0.50	0.44	0.47	0.48	0.50	0.48	0.50
Eulamprus tympanum	0.58	0.53	0.58	0.53	0.68	0.59	0.47	0.51	0.47	0.51	0.62	0.60
Lampropholis delicata	0.51	0.48	0.51	0.48	0.57	0.52	0.39	0.45	0.39	0.45	0.43	0.48
Lissolepis coventryi	0.53	0.46	0.53	0.46	0.53	0.46	0.48	0.48	0.48	0.48	0.48	0.48
Varanus varius	0.47	0.40	0.53	0.43	0.58	0.47	0.35	0.29	0.39	0.32	0.44	0.35

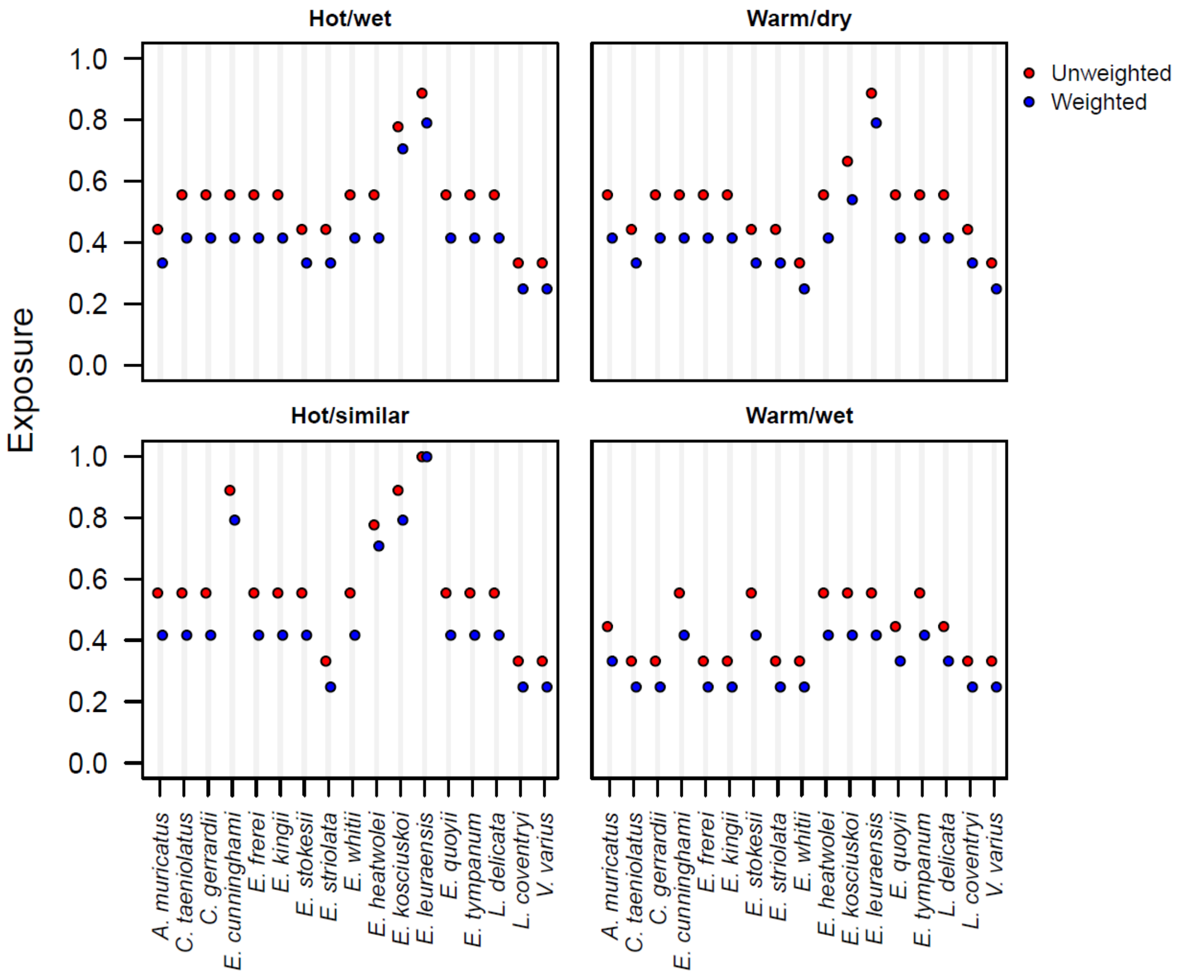

Figure S1: Weighted and unweighted exposure scores for 17 Australian lizards under projected climate change at 2030**.** Exposure was assessed using four contrasting future climate scenarios that are equally plausible: hot/wet; warm/dry; hot/similar precipitation; and warm/wet. Score > 0.67 is high, from 0.33 to 0.67 is moderate and < 0.33 is low.

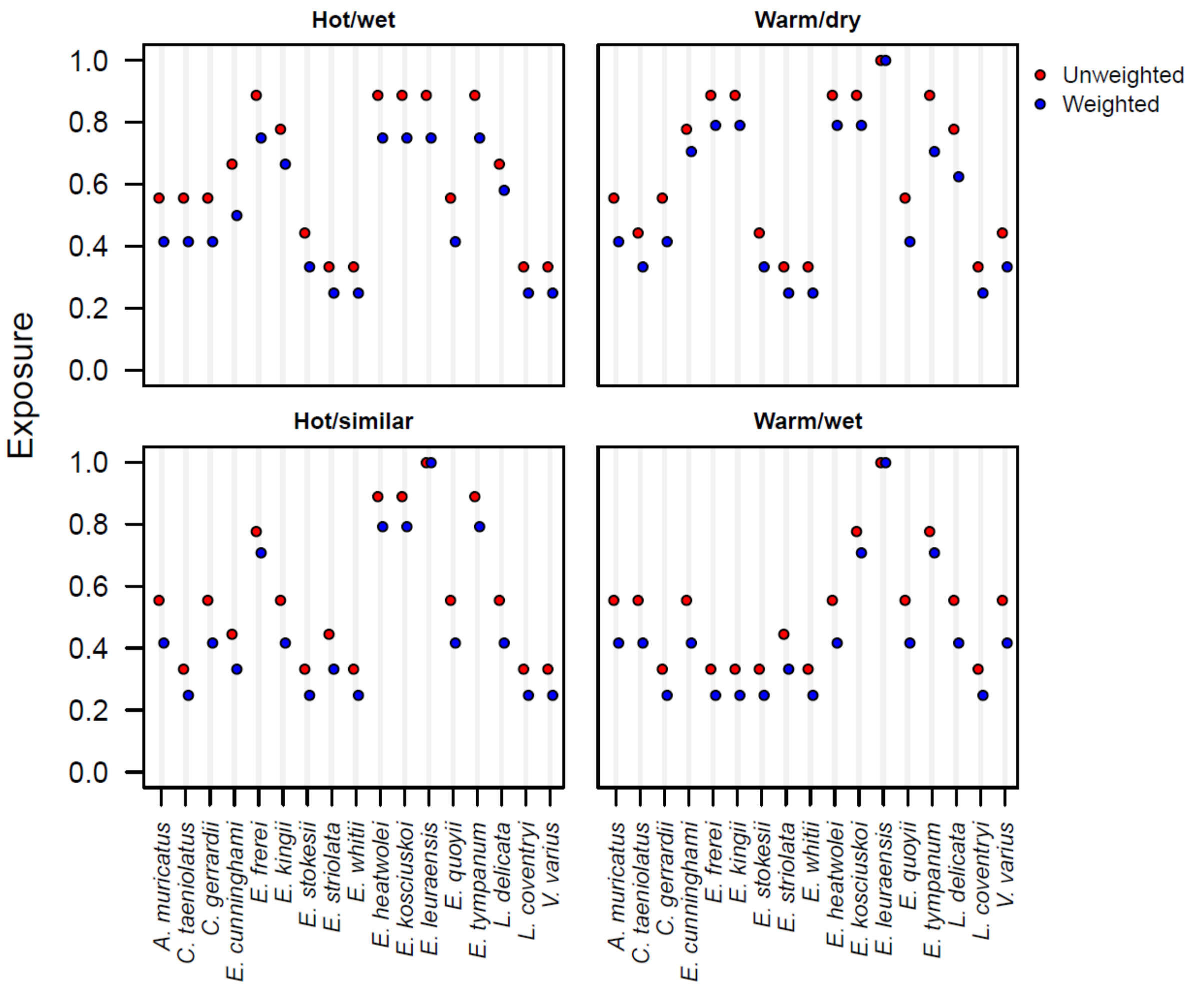

Figure S2: Weighted and unweighted exposure scores for 17 Australian lizards under projected climate change at 2070**.** Exposure was assessed using four contrasting future climate scenarios that are equally plausible: hot/wet; warm/dry; hot/similar precipitation; and warm/wet scenarios. Score > 0.67 is high, from 0.33 to 0.67 is moderate and < 0.33 is low.

References

1. Greer, A. *The Biology and Evolution of Australian lizards*. (Surrey Beatty and Sons, Chipping Norton, Australia, 1989).
2. Light, P., Dawson, W.R., Shoemaker, V.H. & Main, A.R. Observations on the thermal relations of western Australian lizards. *Copeia*, 97-110 (1966).
3. Chapple, D.G. Ecology, life-history, and behavior in the Australian scincid genus Egernia, with comments on the evolution of complex sociality in lizards. *Herpetol. Monogr.* **17**, 145-180 (2003).
4. Greer, A. Critical thermal maximum temperatures in Australian scincid lizards: their ecological and evolutionary significance. *Aust. J. Zool.* **28**, 91-102 (1980).
5. Barwick, R. & Bryant, C. Physiological and biochemical aspects of hibernation in the scincid lizard *Egernia cunninghami* (Gray, 1832). *Physiol. Zool.* 1-20 (1966).
6. Barwick, R.E. *Studies on the scincid lizard Egernia cunninghami (Gray, 1832)*. (Australian National University, Canberra, Australia, 1965).
7. Stow, A., Sunnucks, P., Briscoe, D. & Gardner, M. The impact of habitat fragmentation on dispersal of Cunningham's skink (*Egernia cunninghami*): evidence from allelic and genotypic analyses of microsatellites. *Mol. Ecol.* **10**, 867-878 (2001).
8. Fraser, S.P. Variability of heating and cooling rates during radiant heating in a scincid lizard, *Egernia cunninghami*. *Comp. Biochem. Physiol. A. Physiol.* **80**, 281-286 (1985).
9. O'Connor, D. & Moritz, C. A molecular phylogeny of the Australian skink genera Eulamprus, Gnypetoscincus and Nangura. *Aust. J. Zool.* **51**, 317-330 (2003).
10. Arena, P. & Wooller, R. The reproduction and diet of *Egernia kingii* (Reptilia: Scincidae) on Penguin Island, Western Australia. *Aust. J. Zool.* **51**, 495-504 (2004).
11. Dubey, S., Sinsch, U., Dehling, M.J., Chevalley, M. & Shine, R. Population demography of an endangered lizard, the Blue Mountains Water Skink. *BMC Ecol.* **13**, 1 (2013).
12. Doughty, P. & Shine, R. Reproductive energy allocation and long-term energy stores in a viviparous lizard (*Eulamprus tympanum*). *J. Ecol.* **79**, 1073-1083 (1998).
13. Clemann, N., Chapple, D.G. & Wainer, J. Sexual dimorphism, diet, and reproduction in the swamp skink, *Egernia coventryi*. *J. Herpetol.* **38**, 461-467 (2004).
14. Michael, D. R. *et al.* Ecological niche breadth and microhabitat guild structure in temperate Australian reptiles: implications for natural resource management in endangered grassy woodland ecosystems. *Aust. J. Ecol.* **40**, 651-660 (2015).
15. Schwartz, T., Warner, D., Beheregaray, L. & Olsson, M. Microsatellite loci for Australian agamid lizards. *Mol. Ecol. Notes* **7**, 528-531 (2007).
16. Pepper, M., Barquero, M.D., Whiting, M.J. & Keogh, J.S. A multi-locus molecular phylogeny for Australia's iconic Jacky Dragon (Agamidae: *Amphibolurus muricatus*): Phylogeographic structure along the Great Dividing Range of south-eastern Australia. *Mol. Phylogenet. Evol.* **71**, 149-156 (2014).
17. Hitchen, D., Burgin, S. & Wotherspoon, D. Notes on the size structure of a population of the Jacky Dragon *Amphibolurus muricatus* in a small fragmented urban remnant. *Pac. Conserv. Biol.* **16**, 237-243 (2010).
18. Hitchen, D.J., Burgin, S., Ridgeway, P. & Wotherspoon, D. Habitat use by the jacky lizard *Amphibolurus muricatus* in a highly degraded urban area. *Animal Biol.* **61**, 185-197 (2011).
19. Cogger, H. *Australian Reptiles and Amphibians*. (Reed New Holland, Sydney, Australia, 2000).
20. Harradine, E., How, R., Schmitt, L., de Hass, C. & Spencer, P. Isolation and characterisation of 36 polymorphic microsatellite markers using 454 sequencing in the bar-shouldered skink, *Ctenotus inornatus*. *Conserv. Genet. Resour.* **5**, 207-210 (2013).

21. Dennison, S., Smith, S.M. & Stow, A.J. Long-distance geneflow and habitat specificity of the rock-dwelling coppertail skink, *Ctenotus taeniolatus*. *Aust. J. Ecol.* **37**, 258-267 (2012).
22. Koumoundouros, T., Sumner, J., Clemann, N. & Stuart-Fox, D. Current genetic isolation and fragmentation contrasts with historical connectivity in an alpine lizard (*Cyclodomorphus praealtus*) threatened by climate change. *Biol. Conserv.* **142**, 992-1002 (2009).
23. Langkilde, T., O'Connor, D. & Shine, R. Shelter-site use by five species of montane scincid lizards in south-eastern Australia. *Aust. J. Zool.* **51**, 175-186 (2003).
24. Fuller, S.J., Bull, C.M., Murray, K. & Spencer, R. Clustering of related individuals in a population of the Australian lizard, *Egernia frerei*. *Mol. Ecol.* **14**, 1207-1213 (2005).
25. Gardner, M., Cooper, S., Bull, C. & Grant, W. Brief communication. Isolation of microsatellite loci from a social lizard, *Egernia stokesii*, using a modified enrichment procedure. *J. Hered.* **90**, 301-304 (1999).
26. Gardner, M., Bull, C., Cooper, S. & Duffield, G. Microsatellite mutations in litters of the Australian lizard *Egernia stokesii*. *J. Evol. Biol.* **13**, 551 (2000).
27. Michael, D.R., Cunningham, R.B. & Lindenmayer, D.B. The social elite: habitat heterogeneity, complexity and quality in granite inselbergs influence patterns of aggregation in *Egernia striolata* (Lygosominae: Scincidae). *Aust. J. Ecol.* **35**, 862-870 (2010).
28. Duckett, P.E., Morgan, M.H. & Stow, A.J. Tree-dwelling populations of the skink *Egernia striolata* aggregate in groups of close kin. *Copeia* **2012**, 130-134 (2012).
29. Chapple, D.G. Life history and reproductive ecology of White's skink, *Egernia whitii*. *Aust. J. Zool.* **53**, 353-360 (2006).
30. Chapple, D.G. & Keogh, J.S. Complex mating system and dispersal patterns in a social lizard, *Egernia whitii*. *Mol. Ecol.* **14**, 1215-1227 (2005).
31. Morrison, S.F., Keogh, J.S. & Scott, I.A.W. Molecular determination of paternity in a natural population of the multiply mating polygynous lizard *Eulamprus heatwolei*. *Mol. Ecol.* **11**, 535-545 (2002).
32. Scott, I.A.W., Hayes, C.M., Keogh, J.S. & Morrison, S.F. Isolation and characterization of novel microsatellite markers from the Australian water skink *Eulamprus kosciuskoi* and cross-species amplification in other members of the species-group. *Mol. Ecol. Notes* **1**, 28-30 (2001).
33. Dubey, S. & Shine, R. Restricted dispersal and genetic diversity in populations of an endangered montane lizard (*Eulamprus leuraensis*, Scincidae). *Mol. Ecol.* **19**, 886-897 (2010).
34. Noble, D.W., McFarlane, S.E., Keogh, J.S. & Whiting, M.J. Maternal and additive genetic effects contribute to variation in offspring traits in a lizard. *Behav. Ecol.* **25**, 633-640 (2014).
35. Blomberg, S.P. & Shine, R. Modelling life history strategies with capture–recapture data: evolutionary demography of the water skink *Eulamprus tympanum*. *Aust. J. Ecol.* **26**, 349-359 (2001).
36. Chapple, D.G. & Thompson, M.B. Isolation and characterization of microsatellite loci from the invasive delicate skink (*Lampropholis delicata*), with cross-amplification in other Australian Eugongylus group species. *Conserv. Genet. Resour.* **1**, 55-58 (2009).
37. Guarino, F. Spatial ecology of a large carnivorous lizard, *Varanus varius* (Squamata: Varanidae). *J. Zool.* **258**, 449-457 (2002).
38. Weavers, B. Vital statistics of the lace monitor lizard (*Varanus varius*) in south-eastern Australia. *Vic. Nat.* **105**, 142-145 (1988).
